# Electret Nonwoven Structures for High-Efficiency Air Filtration, Produced Using the Blow Spinning Technique

**DOI:** 10.3390/ma17246038

**Published:** 2024-12-10

**Authors:** Agata Penconek, Natalia Bąkała, Anna Jackiewicz-Zagórska, Artur Małolepszy, Rafał Przekop, Arkadiusz Moskal

**Affiliations:** Faculty of Chemical and Process Engineering, Warsaw University of Technology, 00-645 Warsaw, Poland; agata.penconek@pw.edu.pl (A.P.); natalia.bakala.stud@pw.edu.pl (N.B.); anna.jackiewicz@pw.edu.pl (A.J.-Z.); artur.malolepszy@pw.edu.pl (A.M.); arkadiusz.moskal@pw.edu.pl (A.M.)

**Keywords:** electret, nonwoven structures, MoS_2_, PLA, SiO_2_

## Abstract

This study explores the fabrication of electret nonwoven structures for high-efficiency air filtration, utilizing the blow spinning technique. In response to the growing need for effective filtration systems, we aimed to develop biodegradable materials capable of capturing fine particulate matter (PM2.5) without compromising environmental sustainability. Polylactic acid (PLA) was used as the primary polymer, with the addition of SiO_2_ and MoS_2_ to enhance the fibers’ charge retention and filtration performance. The fibers were charged electrostatically to improve particle capture efficiency. The experimental results showed that fibers containing 5% MoS_2_ exhibited the highest filtration efficiency, surpassing those with SiO_2_, despite MoS_2_ being a semiconductor and SiO_2_ a dielectric. Furthermore, the addition of MoS_2_ improved the filtration efficiency across a range of particle sizes (0.2–1 µm) while maintaining a manageable pressure drop. These findings suggest that incorporating MoS_2_ in electret nonwoven structures can significantly improve filtration performance, making it a promising material for advanced air filtration applications. This study contributes to the development of eco-friendly filtration materials with high performance, essential in reducing exposure to airborne pollutants.

## 1. Introduction

Air pollution caused by solid particles, both of natural origin, such as desert dust or volcanic ash, and anthropogenic ones (e.g., soot aggregates, microplastics), is one of the most significant problems faced by humanity. Every year, nearly 1 million people die prematurely due to exposure to high concentrations of PM2.5 [1]. Prolonged exposure to particulate matter leads to cardiac, respiratory, and neurological problems. It may also cause an increased risk of developing cancer [2,3]. Depending on their size, solid particles can be carried over long distances by the wind, thus affecting the environment and people, not only in the place of origin [4]. Providing adequate protection against particulate matter, either in the form of high-efficiency filters in ventilation systems or high-efficiency filtration materials used in personal protective equipment, is an important issue. Nowadays, in the era of ubiquitous microplastics and the ever-increasing amount of waste produced, when introducing a product to the market, one should ensure that it is made from biodegradable or recyclable waste and that its production has minimal impact on the environment. This also applies to filter materials, especially those used in disposable personal protective equipment. One disposable N95 filtering half-mask contains approximately 11 g of polypropylene and 4.5 g of other polymers [5]. During the COVID-19 pandemic, 129 billion masks were used per month around the world [5]. Kwak and An estimated that, if at least 1% of the masks used had not been properly disposed of, approximately 30–40 tons of plastic would have been released into the environment each month [6].

There are three main techniques for producing fibers and filter materials: melt blowing, electrospinning, and solution blowing. The most popular of them is melt blowing. In this technique, fibers are created by stretching a molten polymer stream in hot air [7,8]. Melt blowing is the most efficient of the three methods. Still, the high temperatures required during the process limit this technique’s applicability to polymers and additives resistant to temperatures of around 200 °C. Due to the need to use low polymer flow rates, the least effective technique is electrospinning. Nonetheless, this technique allows for the production of fibers from natural polymers such as silk, collagen, gluten, casein, or gelatin [9]. A key limitation of electrospinning is the need to use a high voltage. The third technique mentioned—solution blowing—balances the advantages and disadvantages of the two previously described fiber production methods. Solution blowing enables the production of fibers with nanometric diameters (although not as small as in the case of electrospinning) but without the use of a high voltage and with greater efficiency (though not as high as in the case of melt blowing). Additionally, since high temperatures are not used, it is possible to work with polymers and temperature-sensitive compounds. This makes solution blowing a promising technique that can be used to effectively produce biodegradable structures from natural materials modified at the production stage with additional compounds to impart the desired properties. Introducing essential oils of coriander or cumin at the fiber production stage makes it possible to grant the fibers antibacterial properties [10]. The surface of the fibers can be modified using propolis, beeswax, wasabi, Mauka honey, and many other natural additives [11,12,13].

When looking for natural and/or biodegradable polymers to produce filter materials, it should also be borne in mind that the produced filtration structure must be characterized by a high filtration efficiency, with a simultaneous low-pressure drop and long lifetime.

Increasing filtration efficiency can be achieved in several ways. The filtration efficiency will increase as the average fiber diameter decreases, but at the same time, the flow resistance will also increase. A solution that can improve efficiency and, at the same time, slightly increase pressure drop is the incorporation of nanofibers [14]. To increase the filtration efficiency, one could use layered filters, in which each subsequent layer will have a different filtration efficiency [15]. Another method is to introduce an electric charge onto the surface of the fibers [16]. Polarization effects (caused by the uneven electric field in the electret) and Coulomb interactions make it possible to retain neutral and charged particles on the surface of the electret fibers [17]. Since the charge is accumulated on the surface of the fibers, the smaller the average diameter of the fiber, the greater the collected charge will be. Hence, the polarization effect and Coulomb interactions increase while the fibers’ diameter decreases [18]. However, the initial filtration efficiency of electrets is reduced by temperature, humidity, and organic solvents [19,20,21,22]. Electrets tend to lose their charge quickly, so, at the production stage, it is worth using additives to ensure that the charge is retained. This function can be performed, for example, by SiO_2_ and BaTiO_3_ (dielectrics), but also by various nucleating agents (e.g., 1,4-phenylene-bisamides, sodium 2,2′-methylene-bis(4,6-di-tert-butylphenyl)-phosphate, and N,N′-dicyclo-hexyl-2,6-naphthalene-dicarbox-amide), antioxidants, or light stabilizers [23,24,25,26]. Moreover, other additives that cause changes in the fiber structure may also influence charge retention.

Our research aimed to create a high-efficiency filtration structure by blowing from a polymer solution made of biodegradable material containing SiO_2_ and MoS_2_. The process of charging fibers was used to increase the filtration efficiency. The selected additives ensured charge stability on the fiber surface and granted the obtained fibers additional properties.

## 2. Materials and Methods

### 2.1. Solution Blow Spinning

An 8% (*w*/*w*) polylactide PLA (Ingeo™ 6202D, NatureWorks^®^ LLC, Minneapolis, MN, USA) solution in a 3:1 chloroform/acetone (Sigma-Aldrich, Poznań, Poland) mixture was used in the polymer solution-blowing process. The solution was prepared 24 h before the blowing process. The appropriate mass of PLA was placed in a chloroform/acetone mixture in a tightly closed container on a magnetic stirrer.

Depending on the experiment variant, MoS_2_ (2% and 5% [*w*/*w*]) (eLube, Lublin, Poland) and SiO_2_ (0.5%, 1%, 2%, and 5% [*w*/*w*]) (CAS 7631-86-9, Warchem, Zakręt, Poland) were added to the PLA solution in the chloroform/acetone mixture.

**Figure 1:**
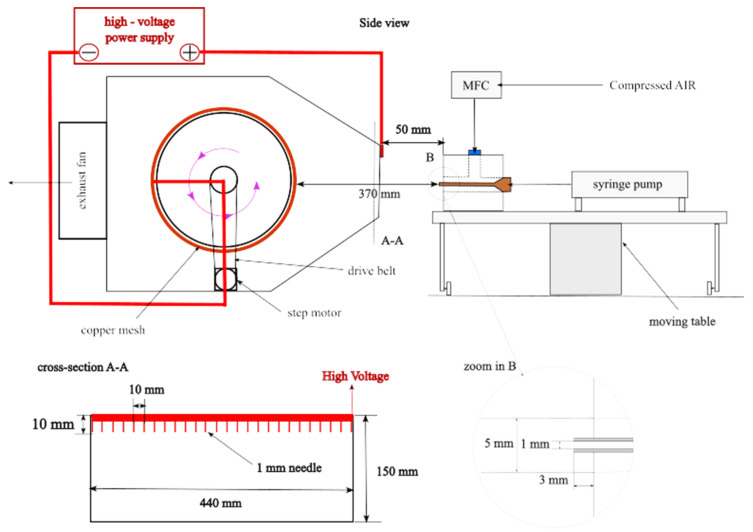
The scheme of the solution-blowing set-up (MFC—mass flow controller).

The apparatus for fiber production and electrostatic charging utilized the conventional solution blow spinning (SBS) technique. The research set-up consisted of the following elements: a syringe pump (Legato270, KDScientific, Holliston, MA, USA) equipped with a 60 mL syringe, a compressed air supply system, a mass flow controller (SFC5500–200 slm, Sensirion, Zurich, Switzerland), a collector, and coaxial nozzles with an internal diameter of 1 mm and an external diameter of 5 mm. A detailed description of the research set-up can be found in our previous works [13]. The system was modified with an advanced fiber-forming head, which was continuously fed with a polylactic acid (PLA) solution via a precision-controlled syringe pump. This head was mounted on a linear actuator that facilitated controlled reciprocating movement. The head-to-collector distance was precisely set to 37 cm. The collector itself was a grounded, copper mesh drum housed in a ventilated enclosure, equipped with an exhaust fan for effective airflow management. At a distance of 5 cm from the nozzle, an array of 44 uniformly spaced needles, each 1 cm in length and separated by 1 cm, were positioned. This needle array was connected to a high-voltage power supply, delivering a stable direct current of 13 kV to induce the necessary electrostatic forces for fiber formation and deposition. The scheme of the research system is shown in Figure 1. Blowing was carried out at an airflow rate of 200 L/min and a 0.5 mL/min polymer flow. The blowing time was 40 min. A minimum of three fibrous structures were created for each tested additive, concentration, and current condition.

### 2.2. Viscosity Measurement

The apparent viscosity of the PLA solution and PLA with additives was determined using an oscillating rheometer (MCR 102, Anthon Paar, Vienna, Austria) equipped with a Peltier system and an attachment limiting the evaporation of solvents in a plate–plate system (50 mm). The test was carried out at a temperature of 22 °C. Each solution was tested at least four times, and the results presented are the average value.

### 2.3. TGA Analysis

Thermogravimetric analysis (TGA) measurements were performed using a TGA/DSC 3+ system (Mettler Toledo, Greifensee, Switzerland). The analysis was performed within a temperature range of 30–1000 °C, at a heating rate of 5 °C/min·, under a synthetic air flux of 60 mL/min (≥99.999%, Multax, Zielonki-Parcela, Poland) to provide an oxidizing atmosphere for the analysis. An aluminum sample holder was used for the measurements.

### 2.4. Fiber Morphology

The morphology of the fibers was determined based on photos from a scanning electron microscope (TM-1000, Hitachi, Tokyo, Japan). Before taking the picture, the fibers were sputtered with a 25 nm layer of gold (K550X EMITECH Quorum, East Sussex, UK). To determine the fiber size distribution and the average fiber diameter, approximately 100 fibers were analyzed.

The produced fibers were also characterized in terms of the porosity of the entire layer and its thickness. The thickness of the produced fiber layer was measured using an optical microscope (PCE-MM, PCE Instruments, Sosnowiec, Poland). The porosity (*α*) of the fiber layer was determined based on the mass of deposited fibers (*m*), the measured thickness of the fiber layer (*L*), the collector area (*A* = 871.5 cm^2^), and the density of PLA (*ρ* = 1.24 g/cm^3^), according to the following relationship:

(1) $$\alpha =\frac{\left(A\cdot L-\frac{m}{\varrho }\right)}{A\cdot L}$$

### 2.5. Experimental Filtration Efficiency and Stability Test

The impact of adding silicon oxide (SiO_2_) and molybdenum disulfide (MoS_2_) particles to PLA on the amount of charge accumulated during the production process was evaluated by assessing the filtration efficiency. The filtration efficiency of the materials in their initial state was analyzed using the Palas MFP 2000 system (Palas GmbH, Karlsruhe, Germany). The primary parameters considered during the design of the filtration materials, specifically filtration efficiency and pressure drop, were evaluated at the beginning of the process.

In the filtration experiments, Arizona Fine Test Dust ISO 12103-1 [27], a polydisperse silica-based test dust (Powder Technology Incorporated, Arden Hills, MN, USA), was used. This dust consists of particles with diameters ranging from 0.2 to 16 μm, with the most common particle sizes falling between 0.35 and 0.45 μm.

The experimental set-up MFP 2000 included the following equipment: a piston-brush particle generator (RBG 1000, Palas GmbH, Karlsruhe, Germany), a pneumatic filter holder, an optical particle counter (PCS 2010, Palas GmbH, Karlsruhe, Germany), a charge neutralizer (CD 2000, Palas GmbH, Karlsruhe, Germany), and a vacuum pump (ASP 2000, Palas GmbH, Karlsruhe, Germany) to collect solid aerosol samples for analysis. Clean air was drawn into a pressurized vessel, where it was filtered and then split into two streams: one directed to the aerosol generator and the other to a mass flow controller. The rotating brush in the generator ensured the uniform dispersion of dust particles within the airstream. Aerosol particles that became electrostatically charged while passing through the brush generator, piston, and connecting tubes were neutralized by mixing the aerosol stream with bipolarly ionized gas. This step was essential to ensure that any electrical charge measured originated exclusively from the intentionally charged fibers in the filter material. The neutralized aerosol was then introduced into a column containing the tested filter material, secured within a pneumatic holder with a circular cross-section of 14 cm in diameter, resulting in an effective test area of approximately 150 cm^2^. The pressure drop across the filter was continuously monitored using a digital differential manometer. Simultaneously, the particle concentration and size distribution in the airflow were measured using an optical particle counter, with equal aerosol samples drawn in by the vacuum pump.

For each tested filter material, both charged and uncharged, three separate measurements of the initial filtration efficiency and pressure drop were conducted. These repeated measurements confirmed the consistency and repeatability of the results. The filtration efficiency values presented in this study represent the average of three samples for each material. All tests were conducted under identical process conditions, including an aerosol flow velocity of 0.2 m/s and a consistent silica particle concentration.

Due to the configuration of the testing apparatus, which was equipped with only one sampling probe and one particle counter, the measurement procedure was as follows. First, the particle concentration in the incoming aerosol stream was measured. Then, the filter material was placed in the pneumatic holder, and the number of particles downstream of the filter was recorded. To verify the stability of the particle generator, an additional measurement of the upstream particle concentration was performed after the filter was removed from the holder. Each measurement lasted for 40 s, and the results confirmed the stability of the silica particle source throughout the tests.

Before conducting the main filtration efficiency tests, an initial investigation was carried out to determine the distribution and adhesion of the added particles within the PLA fibers. Specifically, the aim was to establish whether the particles were encapsulated within the fibers or situated on their surface, and, if on the surface, whether they were securely attached or prone to being re-entrained by the airflow. To address this, a stream of clean air (without any particles) at varying flow velocities was passed through the filter material. The optical particle counter continuously monitored for any particles downstream of the filter. If particles were detected in the air behind the filter during this test, it would mean that the SiO_2_ and MoS_2_ particles were not permanently attached to the fiber and detached from it as the air flowed through the filter. Thus, the filter could not be used in research. The results of this experiment demonstrated that the added particles of SiO_2_ and MoS_2_ remained firmly attached to the surface of the PLA fibers during the airflow.

### 2.6. Theoretical Filtration Efficiency

The theoretical initial filter efficiency was calculated based on classical filtration theory, summarized by Podgórski [28] as follows:

(2) P=1−exp(−λL)

where *L* is the filter media thickness. The filter coefficient, *λ*, is related to single-fiber efficiency, *E*, as follows:

(3) $$\lambda =\frac{4\alpha E}{\pi d_{f}\left(1-\alpha \right)}$$

where *α* is the fiber’s packing density, and *d*_f_ is the fiber diameter. The single-fiber efficiency (defined as a ratio of the flux of particles depositing onto the fiber to the flux of particles passing a surface, being the projection of the fiber onto a plane perpendicular to the direction of mean motion) was calculated assuming that the deposition efficiency due to deterministic mechanisms, *E*_det_ (inertial impaction, interception, sedimentation, and electrostatic forces), and stochastic mechanism (Brownian diffusion), *E_diff_*, was independent:

(4) $$E=1-\left(1-E_{det}\right)\left(1-E_{diff}\right)$$

Particle collection by interception occurs when a particle follows a gas streamline that happens to come within a particle radius from the surface of a fiber. The particle hits the fiber and is captured due to its finite size. The single-fiber deposition efficiency due to interception depends on the dimensionless parameter *R*, defined as follows:

(5) $$R=\frac{d_{p}}{d_{f}}$$

where *d*_p_ is the aerosol particle diameter.

The single-fiber efficiency due to interception, *E*_R_, was given by Lee and Ramamurthi [29] as follows:

(6) $$E_{R}=\frac{\left(1-\alpha \right)R^{2}}{Ku\left(1+R\right)}$$

where the Kuwabara factor, *Ku*, is defined as

(7) $$Ku=-0.5ln\alpha -0.75+\alpha -0.25\alpha^{2}$$

Inertial impaction occurs when the particle, because of its inertia, is unable to adjust quickly to the abruptly changing streamlines near the fiber and crosses those streamlines to hit the fiber. The parameter that governs this mechanism is Stokes number, *Stk*:

(8) $$Stk=\frac{d_{p}^{2}\rho_{p}C_{C}U}{18\mu d_{F}}$$

where *μ* is the fluid viscosity, *ρ*_p_ is the density of the particle sand, and *U* is the superficial velocity of flow.

The Cunningham slip correction factor, *C_c_*, which corrects for reduced drag force on particles in gas flow, is calculated as follows [30]:

(9) $$C_{C}=1+\frac{\lambda_{g}}{d_{p}}\left[2.33+0.966exp\left(-0.4985\frac{d_{p}}{\lambda_{g}}\right)\right]$$

where *λ*_g_ is the mean free path of gas molecules.

The single-fiber efficiency for inertia, *E*_I_, is given by Stenhouse [31]:

(10) $$E_{I}=\left(1+R-E_{R}\right)\left(1-J^{-1}\right)\;for\;J\geq 1$$

(11) $$E_{I}=0\;for\;J<1$$

(12) J=0.45+1.4α+(1.3+0.5logα)Stk

Finally, the deposition efficiency due to inducted forces, *E_in_*, [32] is taken into account:

(13) $$E_{in}={\left(\frac{1-\alpha }{Ku}\right)}^{2/5}\frac{\pi K_{in}}{1+2\pi K_{in}^{2/3}}$$

The inducted force parameter, Kin, is given by the following:

(14) $$K_{in}=\frac{\;2C_{C}Q_{f}^{2}d_{p}^{2}}{3\mu \varepsilon_{0}{\left(1+\varepsilon_{f}\right)}^{2}d_{f}U}\frac{\varepsilon_{p}-1}{\varepsilon_{p}+2}$$

where *Q*_f_ is the charge density on fiber, *ε*_0_ is the dielectric constant of vacuum, and *ε*_f_ and *ε*_p_ are the relative dielectric constants of fibers and particles, respectively.

## 3. Results

### 3.1. Theoretical Filtration Efficiency

**Figure 2:**
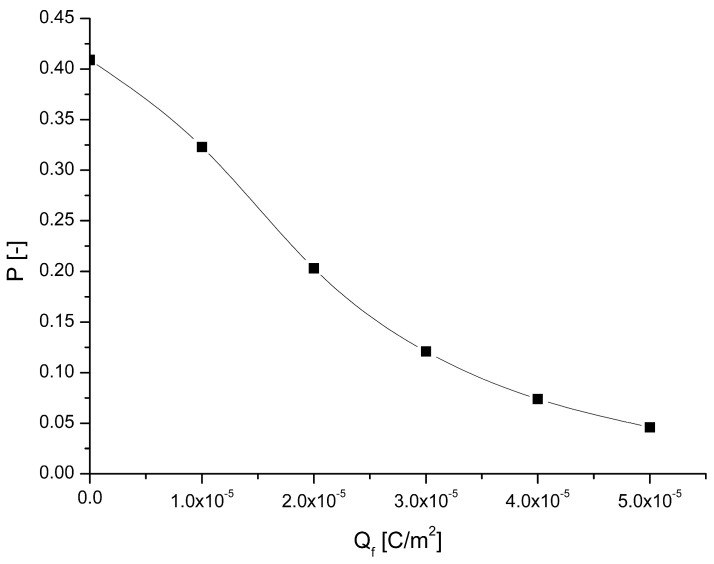
The calculated penetration of particles as a function of surface charge density.

The calculated penetration of particles of diameter 0.25 µm through filter media as a function of fibers’ surface charge density (*Q_f_*) is shown in Figure 2. The charge densities of order 10^−5^ C/m^2^ are typical for electret fibers [33]. The filter structure was assumed to be similar to the PLA filters obtained in previous research [15]. The fiber diameter was 0.8 μm, the packing density 0.95, the filter depth 0.15 mm, and the superficial air velocity 0.2 m/s. The results show that the immobilization of electric charge on the fiber surface leads to a significant decrease in the penetration of submicron particles though filter media even for uncharged particles, when only inducted force is taken into account and Coulomb force is neglected. It is particularly important as, for such particles, neither inertial nor diffusive mechanisms of deposition are effective.

### 3.2. Rheological Properties

**Figure 3:**
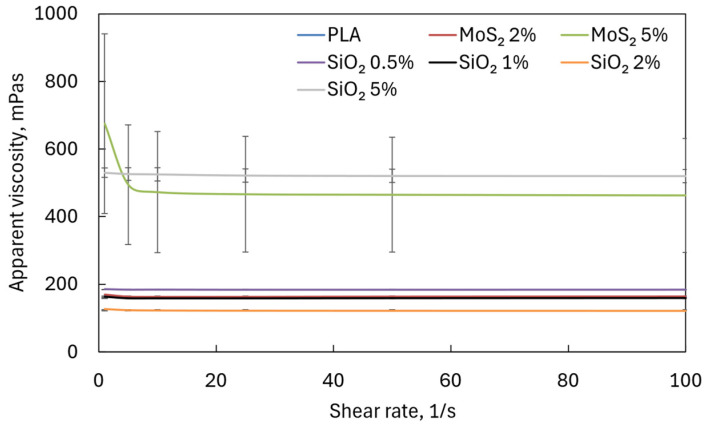
Apparent viscosity of analyzed PLA solution. The curve for pure PLA coincides with the curve for MoS_2_ 2%; therefore, it is invisible in the graph.

All analyzed solutions were non-Newtonian shear-thinning fluids. An increase in the MoS_2_ concentration in the PLA solution led to an increase in the apparent viscosity. In contrast, an increase in the SiO_2_ concentration in the range of 0.5–2% led to a decrease in the apparent viscosity. However, the apparent viscosity of 5% SiO_2_ was higher than the apparent viscosity of pure PLA. Figure 3 shows the relationship between apparent viscosity and shear rate. Due to the scale, the characteristic course of changes in apparent viscosity as a function of shear rate for non-Newtonian shear-thinning fluid is clearly visible only for 5% SiO_2_.

### 3.3. TGA

**Figure 4:**
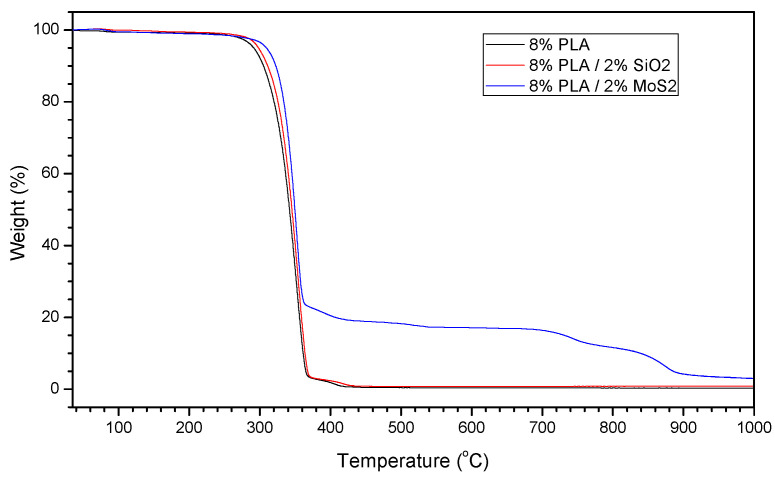
TGA showing the decomposition of PLA and PLA with SiO_2_ and MoS_2_ mixtures.

The TGA of the obtained fibers (Figure 4) showed that samples made from PLA materials began to degrade after exceeding 270 °C, while samples containing SiO_2_ and MoS_2_ began to degrade after 285 °C and 300 °C, respectively. At 360 °C, PLA was completely oxidized, leaving only SiO_2_ and MoS_2_ in the samples. Above 700 °C, MoS_2_ started to sublime. Based on the TGA results, it was observed that, in the PLA/MoS_2_ sample, MoS_2_ constituted 19.0% of the total, and, in the PLA/SiO_2_ sample, SiO_2_ constituted 0.5% of the total. The high MoS_2_ content in the sample might have resulted from MoS_2_ oxidation occurring above 250 °C.

### 3.4. Morphology of Fibers

Fiber materials produced via the polymer solution-blowing process from PLA solutions containing various additives with different concentrations were characterized by their thickness, porosity, and average fiber diameter. The blowing process was carried out each time under the same conditions of airflow, polymer flow, and blowing time; therefore, the differences in the fiber characteristics (average fiber diameter, porosity, and fiber layer thickness) resulted from the use of an external electric field and the properties of the additive used.

**Figure 5:**
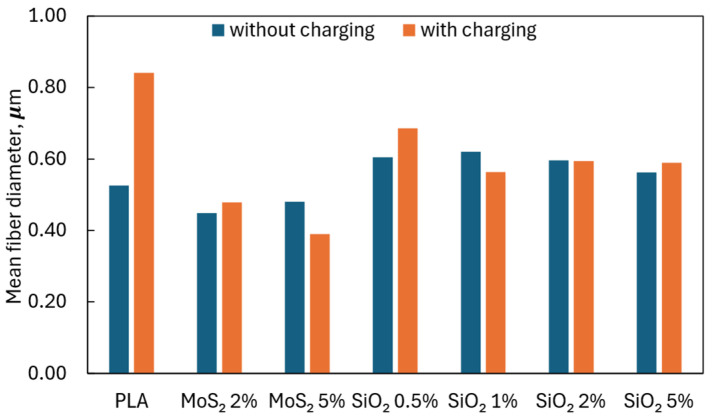
Mean fiber diameter.

It was observed that the addition of MoS_2_ caused a decrease in the average fiber diameter compared to the value obtained for pure PLA, both in the case of fibers charged and uncharged in an electric field. In turn, the addition of SiO_2_ increased the average diameter of the fiber compared to fibers obtained from pure PLA in conditions without an external electric field. The opposite effect was observed when the fibers were charged in an external electric field. The average diameters of charged fibers containing SiO_2_ were smaller than the average diameters of fibers obtained from pure PLA. Average fiber diameters and diameter ranges are shown in Table 1 and Figure 5.

The external electric field did not significantly affect the average fiber diameter within the additives and concentrations used. Only for the pure PLA solution was there almost a two-fold increase in the average diameter of the fibers when an electric field was used.

**Figure 6:**
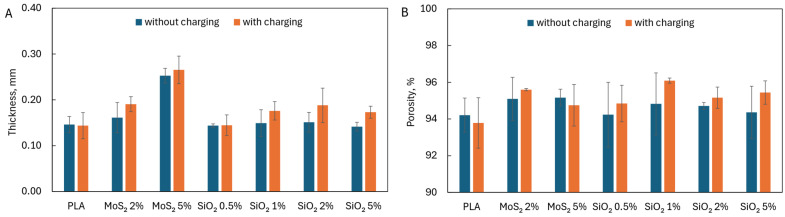
(**A**) Thickness and (**B**) porosity of fiber layers.

Applying an external electric field did not affect the thickness of the produced fiber layer (Figure 6A). Similarly, the use of SiO_2_ (regardless of the concentration) did not increase the thickness of the fiber layer. Only the addition of 5% MoS_2_ caused an almost two-fold increase in the thickness of the fiber layer but did not lead to a significant change in the porosity of the fiber layer (Figure 6B). The porosity of all produced fiber layers was similar (94.88 ± 0.61), regardless of using an external electric field or additives.

**Figure 7:**
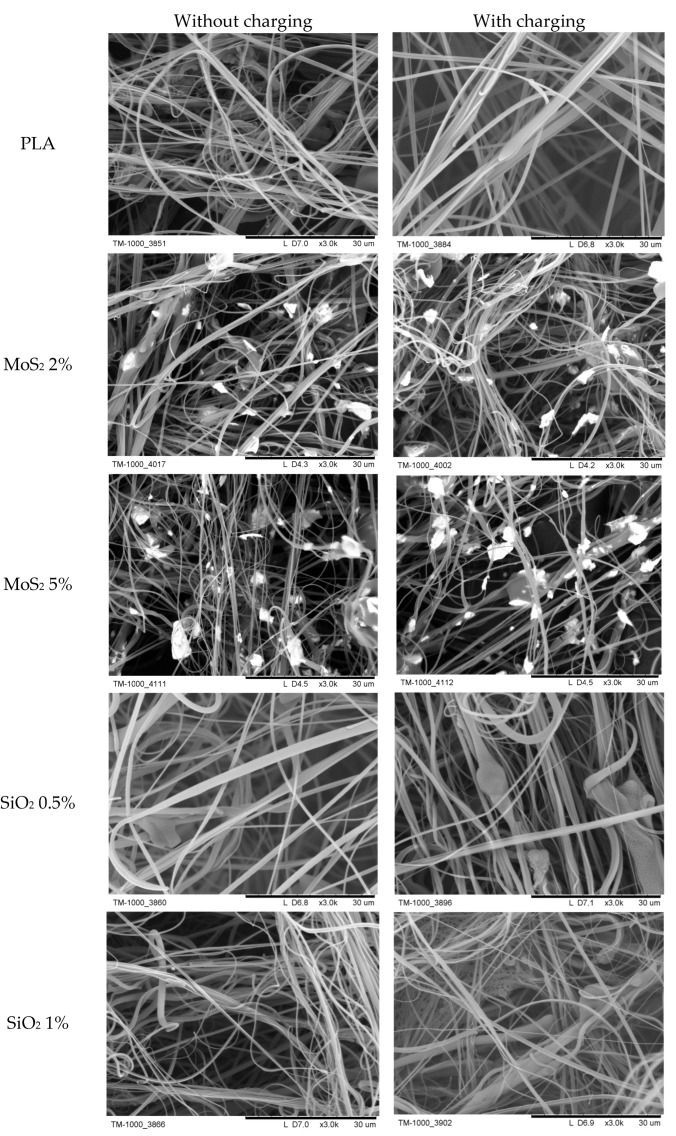

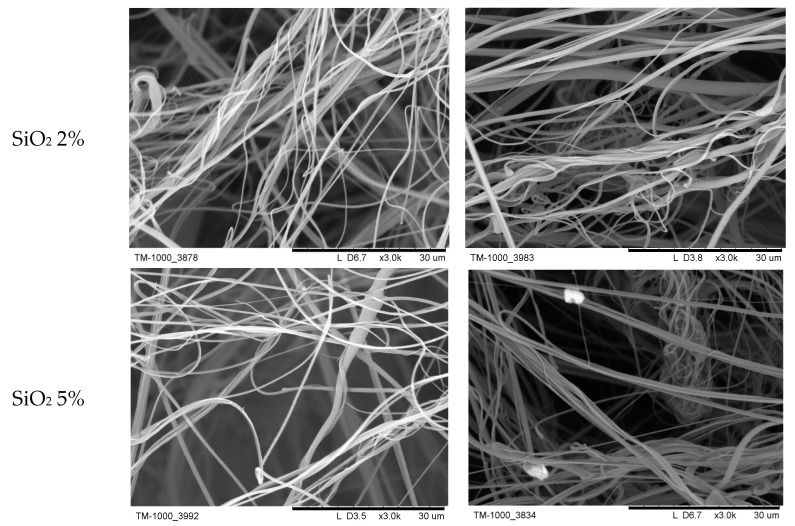
SEM photos of fibers. The units on the scale are in micrometres.

Figure 7 shows SEM photos of sample structures. MoS_2_ particles are visible on the fiber surface. Still, despite more than doubling the MoS_2_ concentration in samples 2% MoS_2_ and 5% MoS_2_, the concentration of particles on the surface of the fibers (qualitatively assessed based on the photo) is at a similar level. SiO_2_ particles are much less present on the fiber surface, but, as in the case of MoS_2_, an increase in the SiO_2_ concentration in the sample does not translate into a higher concentration of SiO_2_ particles on the fibers. These observations are consistent with the results of the TGA, which showed that the SiO_2_ content in the fibers, at the same SiO_2_ and MoS_2_ concentration in the blown sample, was two orders of magnitude lower.

MoS_2_ and SiO_2_ particles on the fiber surface were permanently bonded to it. No detachment of particles from the fiber surface was observed during the test, which involved passing a clean airstream through the produced fiber layer.

### 3.5. Experimental Filtration Efficiency

**Figure 8:**
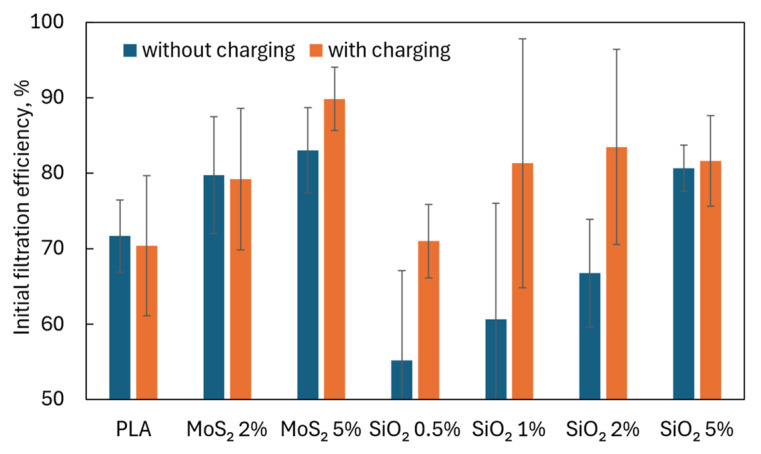
Initial filtration efficiency.

The deposition of particles on the fiber surface occurs due to the interaction of four mechanisms. As the particle diameter increases, the efficiency of its deposition on the fiber surface increases due to direct engagement, inertia, and gravitational interactions. The smaller the particle, the more crucial Brownian motion (diffusion) is in its effective deposition. Electrostatic interactions induced by fiber charging improve the filtration efficiency of particles in the nanometer range. However, the charge must be retained on the fiber surface. Dielectrics such as SiO_2_ are used for this purpose. The tests showed (Figure 8) that the initial filtration efficiency was higher for PLA fibers containing SiO_2_ that had been charged in an electric field than for PLA fibers also containing SiO_2_ that had not been charged in an electric field. However, it could be seen that the increase in the initial filtration efficiency with increasing SiO_2_ concentrations for fibers charged in an electric field reached the limit value for the concentration of 2% SiO_2_. A further increase in the SiO_2_ concentration did not increase the filtration efficiency for fibers charged in an electric field. In the case of fibers containing SiO_2_ not charged in an electric field, an increase in SiO_2_ concentration caused an increase in filtration efficiency, but only for a concentration of 5% SiO_2_ the value of the initial filtration efficiency was higher than in the case of pure PLA. The results of experimental studies are included in Supplementary Materials.

The addition of MoS_2_ (in both concentrations) caused an increase in filtration efficiency above the filtration efficiency of pure PLA. Still, the effect of charging the fibers in an electric field on the rise in filtration efficiency was visible only at a concentration of 5% MoS_2_. The initial filtration efficiency for fibers charged in an electric field for 5% MoS_2_ was even higher than the highest filtration efficiency obtained for SiO_2_, even though SiO_2_ is a dielectric and MoS_2_ is a semiconductor.

**Figure 9:**
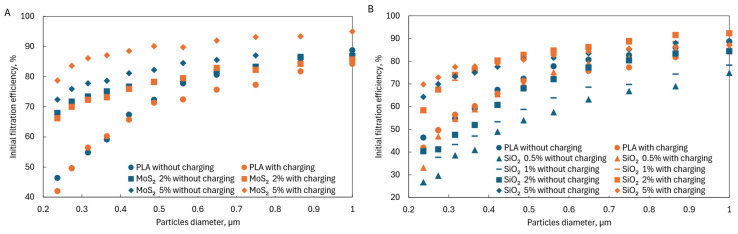
Initial fractional filtration efficiency for (**A**) PLA and PLA-MoS_2_ fibers and (**B**) PLA and PLA-SiO_2_ fibers.

Moreover, fibers with the addition of MoS_2_ also achieved much higher initial fractional filtration efficiencies in the filtered particle size range from 0.2 to 1 µm (Figure 9A). These filtration efficiencies were higher than those achieved by pure PLA fibers, reaching almost twice the filtration efficiency for particles with a size of 0.2–0.3 µm.

For fibers with the addition of SiO_2_, a higher initial filtration efficiency in the particle size range of 0.2–1 µm than for fibers made of pure PLA was achieved in the case of fibers charged in an electric field containing 1%, 2%, and 5% SiO_2_ and 5% SiO_2_ without charging (Figure 9B). Compared to pure PLA fibers, the maximum increase in filtration efficiency was 1.5 times greater.

## 4. Discussion

**Figure 10:**
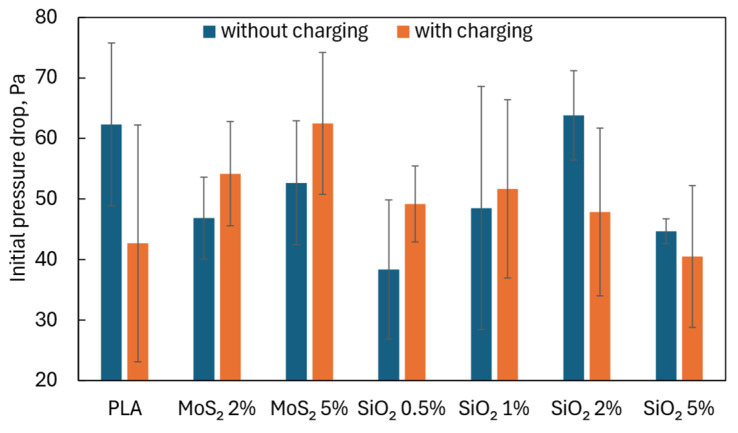
Initial pressure drop.

A high filtration efficiency is desirable, but often, if achieved due to reduced porosity, increased thickness of the filtration structure, and a significant reduction in the average fiber diameter, it is accompanied by a high pressure drop when air flows through the tested structure. The fibrous layers containing additives produced by us were characterized by a pressure drop that did not exceed the pressure drop for fibers made of pure PLA (Figure 10). Higher pressure drops were recorded for fibers with the addition of MoS_2_ than for fibers with the addition of SiO_2_.

**Figure 11:**
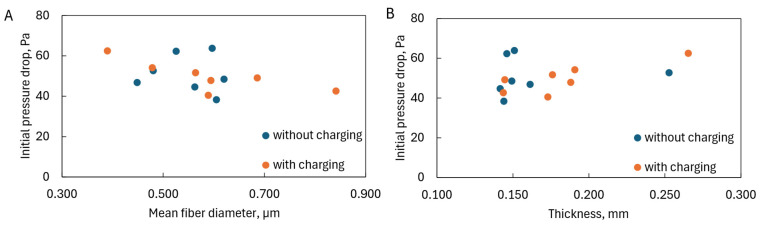
Initial pressure drop as a function of (**A**) mean fiber diameter and (**B**) thickness of fiber layer.

The pressure drop increased with decreasing average fiber diameters (Figure 11A) but was not affected by the presence of charges on the fiber surface. However, due to the similar porosity of the structures produced, the pressure drop did not change with their thickness (Figure 11B).

**Figure 12:**
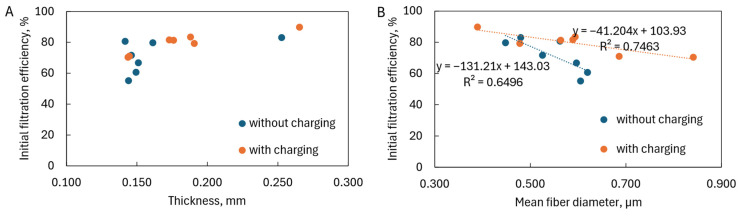
Initial filtration efficiency as a function of (**A**) thickness of fiber layer and (**B**) mean fiber diameter.

The thickness of the fiber layers also did not affect the initial filtration efficiency (Figure 12A). According to filtration theory, it was influenced by the average fiber diameter: the thinner the fiber, the higher the filtration efficiency (Figure 12B). However, it was visible that, in the case of charged fibers, the filtration efficiency increased slower with a decrease in fiber diameter than in the case of uncharged fibers. The small angle of inclination of the fitting lines for electrets indicated that electrostatic interactions had a much more significant impact on filtration efficiency than the average fiber diameter. Increasing the average fiber diameter, which reduced the filtration efficiency, was compensated in electrets by electrostatic interactions, increasing the deposition of particles on the fibers. However, for filters without charging, the angle of inclination of the curve was significant, and even a small change in the average fiber diameter caused a substantial reduction in filtration efficiency. Unfavorable fitting lines and corresponding low R^2^ values were characteristic features of solution blowing, a susceptible and multi-parameter process.

**Figure 13:**
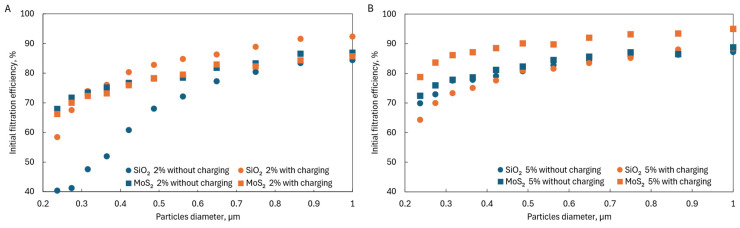
Comparing fractional filtration efficiency for (**A**) 2% MoS_2_ and 2% SiO_2_ and (**B**) 5% MoS_2_ and 5% SiO_2_.

The tests showed that adding MoS_2_ and SiO_2_ affected the filtration efficiency of particles in the range of 0.2–1 µm. In the case of SiO_2_, there were optimal concentrations above which no significant improvement in filtration efficiency was observed; in the case of MoS_2_, there were too few concentrations analyzed to identify such a relationship. When comparing both additives at the same concentrations, it could be seen that higher filtration efficiencies were achieved for MoS_2_ than for SiO_2_ at the same concentrations (Figure 13A,B), which may indicate that MoS_2_ is a better additive.

The addition of MoS_2_ to PLA fibers resulted in a higher filtration efficiency compared to fibers containing SiO_2_. This could be attributed to several key properties of MoS_2_: (i) Among these were its layered structure and high surface area-to-volume ratio. MoS_2_ has a characteristic two-dimensional structure, which provides a large specific surface area. This feature enhances filtration efficiency by improving particle capture. Studies have shown that layered materials, such as MoS_2_, can significantly improve filtration properties due to their increased active surface area [34]. (ii) MoS_2_ addition to the PLA solution resulted in thinner fibers. The layered structure of MoS_2_ allowed its particles to align parallel to the direction of the stretching flow, potentially reducing flow resistance and facilitating the formation of thinner fibers, which increased the active surface area for the filtration process [35]. In contrast, SiO_2_ has a three-dimensional (3D) and often spherical or irregularly shaped structure, which can lead to an increased solution viscosity and the formation of thicker fibers. Additionally, MoS_2_, due to its 2D structure, exhibited minimal agglomeration and better dispersion within the polymer solution, allowing for a lower viscosity even at higher additive concentrations. In contrast, SiO_2_, especially in nano form, tends to aggregate within solutions, which can increase viscosity and hinder the stretching of the flow during fiber formation. (iii) Another important aspect were electrostatic interactions and polarization in an electric field. As a semiconductor, MoS_2_ can undergo polarization in the presence of an electric field, leading to localized changes in charge distribution. These dynamic interactions can enhance the attraction and retention of contaminant particles, thereby increasing filtration efficiency [36]. (iv) Finally, increased electron mobility and surface conductivity were also observed. MoS_2_ exhibited high electron mobility, allowing for a better response to the electric field and the formation of localized electrostatic effects on the fiber surface. This facilitated a more effective attraction of contaminant particles. Studies on composites containing MoS_2_ have demonstrated that its conductive properties can improve filtration efficiency [37].

In summary, the higher filtration efficiency of PLA fibers with MoS_2_ addition resulted from the synergy of its unique structural and electrostatic properties, making it a more effective additive compared to SiO_2_. In addition, MoS_2_ also has increased sorption properties compared to oil [38], so using it for fiber production can grant said fibers additional desirable properties.

## 5. Conclusions

The results show that the presence of MoS_2_ (semiconductor) particles on the surface of PLA fibers obtained in the solution-blowing process significantly increased the filtration efficiency without generating a significant increase in airflow resistance. An increase in filtration efficiency was observed in both electrets and nonwoven filters. The achieved values of the initial filtration efficiency were higher than the filtration efficiency of filters and electrets containing SiO_2_ particles (dielectric).

These findings suggest that MoS_2_ particles are promising additives for producing eco-friendly materials for advanced air filtration applications.

## Figures and Tables

**Table 1:** Average fiber diameters and diameter ranges.

	Mean Fiber Diameter, µm		Diameter Range, µm	
	Without Charging	With Charging	Without Charging	With Charging
PLA	0.525	0.841	0.162–3.081	0.140–4.445
MoS_2_ 2%	0.448	0.478	0.156–2.295	0.144–1.987
MoS_2_ 5%	0.480	0.390	0.143–1.893	0.219–1.157
SiO_2_ 0.5%	0.605	0.686	0.125–2.528	0.165–2.496
SiO_2_ 1%	0.620	0.564	0.145–4.510	0.184–2.404
SiO_2_ 2%	0.596	0.594	0.178–2.505	0.164–3.214
SiO_2_ 5%	0.562	0.589	0.184–2.016	0.173–2.558

## Data Availability

The raw data supporting the conclusions of this article will be made available by the authors on request.

## Supplementary Materials

The supporting information can be downloaded at: www.mdpi.com/article/10.3390/ma17246038/s1.
